# Watching More Closely: Shot Scale Affects Film Viewers’ Theory of Mind Tendency But Not Ability

**DOI:** 10.3389/fpsyg.2017.02349

**Published:** 2018-01-17

**Authors:** Brendan Rooney, Katalin E. Bálint

**Affiliations:** ^1^School of Psychology, University College Dublin, Dublin, Ireland; ^2^Tilburg Center for Cognition and Communication, Tilburg University, Tilburg, Netherlands; ^3^Institute for Media, Knowledge and Communication, University of Augsburg, Augsburg, Germany

**Keywords:** theory of mind, shot scale, close up shot, facial expression, characters, film

## Abstract

Recent research debates the effects of exposure to narrative fiction on recognition of mental states in others and self, referred to as Theory of Mind. The current study explores the mechanisms by which such effects could occur in fictional film. Using manipulated film scenes, we conducted a between subject experiment (*N* = 136) exploring how film shot-scale affects viewers’ Theory of Mind. Specifically, in our methods we distinguish between the trait Theory of Mind abilities (ToM ability), and the state-like tendency to recognize mental states in others and self (ToM tendency). Results showed that close-up shots (compared to long shots) of a character was associated with higher levels of Theory of Mind tendency, when the facial expression was sad but not when it was neutral. And this effect did not transfer to other characters in the film. There was also no observable effect of character depiction on viewers’ general Theory of Mind ability. Together the findings suggest that formal and content features of shot scale can elicit Theory of Mind responses by directing attention toward character mental states rather than improving viewers’ general Theory of Mind ability.

## Introduction

Theory of Mind (ToM), the psychological process by which people recognize and understand the mental states of others is arguably the most important process to human social functioning (Premack and Woodruff, 1978; Tomasello, 1999; Frith, 2012). Supporting this idea, marked social difficulties have been associated with deficits in ToM ability (Baron-Cohen et al., 2001; Shamay-Tsoory et al., 2010) and a low level of mind perception is associated with dehumanization or stigmatization of others (Cameron et al., 2015). Researchers distinguish between the representation of thoughts (cognitive ToM), feelings (affective ToM) and motivations (intentional ToM) of the other (e.g., Dziobek et al., 2008; Shamay-Tsoory et al., 2009). A large body of work also links ToM and related social cognition processes with understanding mental states in the self (Gallese, 2003; Decety and Sommerville, 2003; Neal and Chartrand, 2011; Erbas et al., 2016), further demonstrating the importance of ToM skills. Given the high social value of ToM, researchers are particularly interested in ways to elicit ToM and foster interpersonal sensitivity (Meyer and Lieberman, 2016). Recently, it has been proposed that engagement with narrative fiction is particularly effective in this regard. Drawing on a well-established body of research that identifies the significance of facial-cues in social cognition (e.g., Ekman and Friesen, 1971; Baron-Cohen et al., 2001; van Kleef, 2009), we predict that manipulating film viewers’ visual access to such social cues via shot scale in fictional film narrative will affect ToM response toward characters. We use a true experimental design to explore how shot-scale affects viewers’ ToM. By embedding our research in the everyday act of natural film-viewing, this study offers a high-level of experimental control and a high-level of ecological validity; two typically conflicting characteristics that have been difficult to resolve in research to date.

Narrative fiction has high potential for evoking ToM responses (Mar et al., 2006). Research has demonstrated that high quality literary fiction (Kidd and Castano, 2013, 2016, 2017; Pino and Mazza, 2016), cinematic fiction (Black and Barnes, 2015) and narrativized video-games (Bormann and Greitemeyer, 2015) can improve ToM performance. These findings, however, seem to be difficult to replicate (Panero et al., 2016; Pino and Mazza, 2016), which may be a symptom of the fact that little is known about the mechanisms (in the viewer or the media) that facilitate the increase in ToM. Researchers draw on the work of Mar and Oatley (2008) and propose that ToM performance was superior because the fictional narratives elicited mental simulation and abstraction of social experience. They attribute the ToM performance effects to the effort involved in constructing a mental model of the characters. If this is true then it is reasonable to predict that features of the media may challenge or guide the construction of mental models and differentially affect ToM.

A growing body of work shows that audio–visual narratives are of special importance in eliciting ToM (see Levin et al., 2013; Tan, 2013). One of the main advantages of film over other media is the central role of faces in telling the story. The visual cues carried within human faces are strongly associated with ToM response (Calder et al., 2002; Mosconi et al., 2005; Itier et al., 2007; Itier and Batty, 2009; Fischer et al., 2012). For example, facial expressions and gaze direction are salient triggers of ToM (Frischen et al., 2007). van Kleef’s (2009) Emotions as Social Information (EASI) model explains the link between emotional expression and the observer’s response via inferential and affective reactions. Within this framework, numerous studies have demonstrated the effects of emotional expressions on viewers’ character judgments (Hareli and Hess, 2010), attributions (van Doorn et al., 2015), and inferences about intentions (van Kleef et al., 2004; de Melo et al., 2014). Specific expressions (such as sadness or fear) include social information that tells a story to the viewer (Parkinson, 1999, 2001; Hareli and Hess, 2010). Testament to the importance of reading facial expressions in narrative, Cutting and Armstrong (2016) demonstrate that filmmakers use longer durations for scenes that present faces at a distance, amongst clutter, and argue that this is because viewers need more time to successfully read character expression in a cluttered context. This demonstrates the formal features, such as shot-scale, play an important role in mediating social information in a film.

Shot-scale, defined as the apparent distance of characters from the camera, is one of the most effective visual devices in regulating the relative size of characters’ faces, the relative proportion of the human figure to the background (Salt, 1992; Bowen and Thompson, 2013), and arranging film content according to its saliency (Carroll and Seeley, 2013). It has an impact on self-reported arousal (Canini et al., 2011), prosocial behavior (Cao, 2013), and character liking (Mutz, 2006). Previously, Bálint et al. (2016) observed a relationship between ToM responding and shot-scale distribution within a film. This study found that films with a higher proportion of closer shots (compared to films with fewer or no close shots) evoked higher levels of ToM responding. While the study statistically controlled for various potentially confounding variables, the test condition stimuli were different films (different stories, with different characters). Thus the study was subject to the typical trade-off between experimental control and ecological validity that has been common in the previous research to date. To overcome this limitation, by working with professional animation designers and filmmakers, the present study manipulates shot-scale (by inserting specifically designed close-up shots) into a film, while holding all other variables constant.

Previous research showing a relationship between shot-scale and ToM have failed to clarify if ToM is specifically targeted toward the character who is shown in close-up. It may be reasonable to predict that showing a close-up of a character would elicit ToM toward that character exclusively, yet previous research seems to claim that engagement with fiction results in a non-specific activation of ToM (e.g., Kidd and Castano, 2013; Black and Barnes, 2015). In that case, we would see a transfer effect of target character close-ups on ToM responses toward non-target characters. Thus the present study distinguishes between references to mental states of the target character (who featured in the close-ups) and a non-target character (a character who is seen only in extreme or very long-shots).

Previous studies exploring the effect of narrative fiction have primarily used tasks that explicitly require participants, in a forced-choice test, to identify emotional states (Reading the Mind in the Eyes test; Baron-Cohen et al., 1985), thoughts (Yoni task; Shamay-Tsoory and Aharon-Peretz, 2007; Kalbe et al., 2010) or beliefs (False-belief task; Wimmer and Perner, 1983; Baron-Cohen et al., 1985) from faces or descriptions of scenarios (Happé, 1993, 1994). While these measures have been widely and reliably used for decades (e.g., see Wellman et al., 2001; Fernández-Abascal et al., 2013; Devine and Hughes, 2016), they prompt ToM by explicitly asking about mental states (Apperly and Butterfill, 2009; Rosenblau et al., 2015). The nature of these tasks allows them to successfully tap into participants’ ToM ability (or competence). It has been argued that beyond one’s *ability* to understand mental states, people demonstrate individual differences in their *tendency* to do so, resulting in a ‘competence–performance gap’ (e.g., Meins et al., 2014). Unlike recent distinctions between explicit and implicit ToM, that concern a person’s conscious awareness of their deliberate efforts to mentalize, the distinction between ability and tendency concerns the extent to which a person is prompted or spontaneously models the mental states of another. Apperly (2012) argues that when exploring ToM we must recognize the distinction between the *ability* to conceive of the mind of the other, the mental processes involved in doing so, and the *tendency* to pay attention or care about the mind of the other. Prompting tasks are less sensitive to the absence of mental state references, and are less valid representations of individual differences in adults’ spontaneous ToM (Meins and Fernyhough, 1999). This calls for the use of a measure of ToM-tendency, without which we can say little about unprompted social cognition in everyday life.

Addressing these abovementioned issues, this study employed a data collection method that distinguishes TOM-tendency and TOM-ability (Bálint et al., 2014). It also allows us to break ToM down further by coding whether the participant is mentalizing the character’s cognition, emotion and intentions. Previous studies demonstrated that emotional and cognitive processes of social cognition are interdependent but separate mechanisms in the brain (Dziobek et al., 2008; Zaki and Ochsner, 2012). Therefore, our coding system differentiated whether the theory of mind response referred to cognitive, emotional or intentional mental states in the character. Our procedure was informed by standardized assessments of ToM processes using story-based stimuli and qualitative data collection (Heavey et al., 2000; Dziobek et al., 2006; Golan et al., 2006; Barnes et al., 2009; Dodell-Feder et al., 2013). We are also interested in exploring the way in which character depiction affects references to one’s own mental states (hereafter referred to as ToM-self). This is particularly interesting in light of recent research showing that reading fiction does not elicit a shared emotional state with the characters (Pino and Mazza, 2016).

Our over-arching research question asks how shot-scale affects ToM, that is, the degree to which viewers perceive film-characters as intentional agents with mental states. To partition effects of shot scale from the content of the shot, we also manipulate facial expression of the character in the shot. We refer to these formal and content aspects of shot scale together as “character depiction.” The main research question has three parts: we examine the effect of character depiction on ToM-tendency (RQ1), on ToM-ability (RQ2), and on ToM-self (RQ3). In all cases we predict that close ups increase ToM responses compared to long shots, and this effect will be more pronounced when the target is depicted in a close up with a sad facial expression compared to a neutral facial expression. The use of additional facial expressions may lead to interesting results in the context of the current study, but would each require an additional experimental group in the research design (and thus more participants). As an initial exploration, we use a sad facial expression due to its strong congruence with the major themes of separation in the film, the accompanying music and because a sad expression tends to signal affective tendencies in the observer (Knutson, 1996; Hess et al., 2000; Hareli and Hess, 2010). Aside from testing our main hypotheses, for RQ1 and RQ2, we predict that ToM responses will be higher for the target character than for a non-target character, exploring any possible transfer effects from target to non-target characters.

## Materials and Methods

### Overview

The present study was an online experiment (Qualtrics software) with an incomplete mixed-design. Shot-scale of character (Long-shot vs. Close-up) and Facial expression (Sad vs. Neutral) were levels to the between subject variable collectively referred to as “Character Depiction.” The incomplete design was necessary because facial expression can be only manipulated in close-up condition but not in long shot, where character faces are not seen. The study design also included Character (Target vs. Non-target) as a within subject variable. ToM-tendency and ToM-ability were dependent variables.

### Participants

Power analysis called for a sample between 117 and 141 so as to achieve sufficient power (0.9; α = 0.05) to detect medium effect sizes. Recruiting through a university student participant pool, 170 people started the experiment; 26 of them did not complete the outcome measures and so could not be included in the study. Four participants were excluded due to excessively long duration with the stimulus (>6.5 min) indicating that they did not progress through the study in line with other participants (e.g., rewatching the video or engaging in other tasks). In addition, 2 participants were excluded for reporting to have seen the whole film before and 2 for reporting that they write English at an intermediate level or lower, as this may have affected their ability to express their ToM response (all other participants reported very good, fluent or native-speaker English abilities). Thus the final sample consisted of 136 participants (78 female, 34 male, 24 did not report gender; age: *M* = 22.06, *SD* = 8.71).

### Stimulus Material

We used the first two sequences (2 min) of the multi-international-award winning animated film *Father and Daughter* (Dudok de Wit, 2001) with two characters, a man (non-target character) and a girl (target character). This segment included the title screen “*Father and Daughter*” and credits. The film is a two-dimensional hand-drawn animation, created in a simplistic style, characterized by a limited color palette and simple lines [see Bateman (2014) and Suckfüll (2010) for a formal analysis of the film and responses toward moments of narrative impact]. The film is accompanied by instrumental music (Waves of the Danube), but it contains no dialog or lyrics. The first sequence presents a man (non-target character) and a girl (target character) riding bicycles through a landscape. They arrive to a tree at a lake where the man gets off the bicycle. The girl stops and gets off her bicycle too. The man walks down to the water to a boat, then returns to hug the girl. He walks back to the water, sits into the boat and rows away. The girl stays there standing and watches him rowing away. In the second sequence the girl is again on the same road riding her bicycle. She stops at the same tree, looks at the water, and after a moment she leaves again.

**FIGURE 1:**
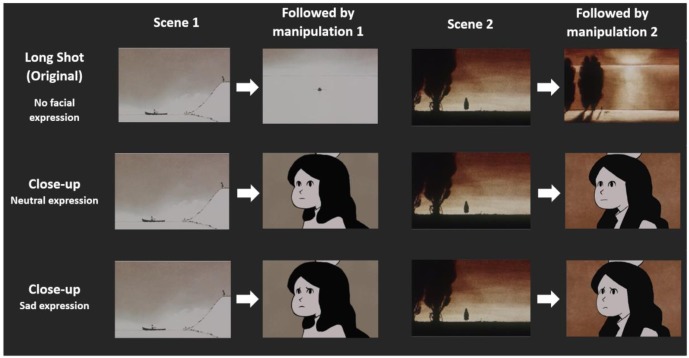
Images of character depiction by condition. Each row depicts an experimental condition. Participants watched a sequence containing two scenes. Depending on the condition, the shot presented in the first and third column (Scenes 1 and 2) was followed by a different shot. Condition 1 (top row) = Long shot is followed by another long shot with no visible facial expression; Condition 2 (middle row): Long shot is followed by a close up shot with neutral facial expression; Condition 3 (bottom row): Long shot is followed by a close-up shot with sad facial expression.

To manipulate the depiction of the target character (shot-scale and facial expression) we developed three different versions of the film excerpt (original version in long-shots, and two manipulated versions in close-ups). The first sequence presents the target character in a point of view shot as she looks at the man rowing away; the second point of view shot at the end of the second sequence presents her again as she looks at the water. In the manipulated versions of the film this long-shot (see **Figure 1**) was replaced by a close-up of the target character with either a sad or neutral facial expression (see **Figure 1**). Animation designers created and edited these close-up shots into the film to be a perfect fit to the style of the original artwork. The length of the films and close-up shots were kept constant. In all conditions the non-target character was depicted in long shots.

In a pilot study we tested the designed close-up shots for emotionality to make sure that the faces were perceived as neutral or sad. Thirty-one participants (15 females; 24 – 38 years old, *M* = 31:28; *SD* = 3.96 years) rated the test faces, after they were given some minor context. The faces were randomly selected by Qualtrics online survey designer, and presented in the order they would appear during the film. For each face, participants had to estimate the age of the depicted character (this is relevant to the narrative), and rate the perceived intensity of discrete emotions (i.e., emotionless, happy, sad, angry, disgusted, fearful, other emotion) on a 9-point scale from “not at all” to “very much.” For each face, the average ratings on each emotion were calculated, these were then combined by group to give a group average rating on each emotion. Comparison of the mean ratings for each group showed that neutral faces evoked significantly higher ratings than sad faces on the dimensions of emotionless, *t*(21.91) = -5.65, *p* < 0.05, *CI_95_* = -4.64, -2.15, and happy, *t*(29) = -2.21, *p* < 0.05, *CI_95_* = -1.42, -0.056; and significantly lower ratings on dimensions of sad, *t*(29) = 5.70, *p* < 0.05, *CI_95_* = 1.71, 3.62, angry, *t*(29) = 5.63, *p* < 0.05, *CI_95_* = 1.49, 3.21, disgust, *t*(29) = 3.12, *p* < 0.05, *CI_95_* = 0.51, 2.42, and fear *t*(29) = 4.98, *p* < 0.05, *CI_95_* = 1.52, 3.64.

### Procedure

The study was approved by the University College Dublin Research Ethics Committee. Participants were asked to complete the experiment in one sitting in an undistracted environment. First they reported their proficiency in the English language; then they were randomly assigned to one of three conditions (Long-shot, Sad close-up or Neutral close-up). After the film, participants responded to three open-ended questions (see **Table 1**). The first question asked participants to describe the story and was designed to allow for ToM-tendency responses. The second question was designed to capture ToM-ability using a prompt to describe the story from the target character’s perspective. Finally, we prompted participants to describe their own experience so as to capture ToM-self. These questions were carefully designed to allow us to explore various ToM effects while minimizing demands on the participants. For example, we decided to use the same question to explore manipulation effects on both target and non-target characters. Once participants responded to these, they completed quantitative control measures of their experience and answered questions about their demographics. At the end of the session, participants were debriefed. Mental state references were assessed using a quantitative content-analytic method by a trained coder, blind to the experimental conditions, developed in prior work (Bálint et al., 2014, 2016) and detailed in the next section. For each of the coded dependent variables, a randomly selected ten percent of descriptions was coded by another independent rater. Agreement was calculated for each variable using Krippendorf’s Alpha; these yielded acceptable levels of agreement (α = 0.67 to 1).

**Table 1:** Description of questions used after viewing and the nature of ToM that they access.

Theory of Mind measures	Question
ToM-tendency	Q.1 Implicit question for unprompted ToM
	*Please describe the story of the film scene in as much detail as possible using at least 6–10 sentences.*
ToM-ability	Q.2 Explicit question prompting ToM for character
	*Try to imagine the story from the perspective of the female character, how would you describe her feelings, thoughts, and intentions? Please write at least 6–10 sentences.*
ToM-self	Q.3 Explicit question prompting ToM for self
	*Describe your own experience during the movie. What happened to you while watching it? How would you describe your thoughts and feelings? Please write at least 6–10 sentences.*

### Measures

#### ToM-Tendency

To measure ToM-tendency we coded responses to question 1, identifying where participants made explicit reference to a mental state. These mental state references were also categorized as referring to the target (female) or the non-target (male) character, and by type of mental state (affective, cognitive or intention; see **Table 2**). Once coded, each participant’s response was given a score for the frequency of mental state references, where higher scores are indicative of higher levels of ToM-tendency in a category.

**Table 2:** Coding frame used to assess frequency of mental state references.

Mental state type^∗^	Reference to…	Example
Affect	Wishes, desires, or feelings	‘Anxious,’ ‘excited,’ ‘feeling lonely’
Cognition	Memory function	‘Forget,’ ‘remember,’ ‘was reminded’
	Knowledge	‘Realize,’ ‘understand,’ ‘assume’
	Other cognition/metacognition	‘Imagine,’ ‘accept,’ ‘pretend’
Intention	Expressed by an explicit word	‘Intend,’ ‘determined to,’ ‘attempt’
	Expressed by a preposition	‘To,’ ‘so that,’ ‘in order to’
	Expressed by a modal verb	‘Have to,’ ‘must,’ ‘want’

*^∗^References to mental states of target (female), non-target (male) and self were coded separately.*

#### ToM-Ability

The ability to use ToM was assessed by coding mental state references occurring in answers to question 2 (which prompted ToM). Again all utterances were coded for explicit references to character mental states and categorized by character (target/non-target) and by type. Higher scores mean more frequent references to mental states, indicating a higher level of ToM-ability.

#### ToM-Self

References to one’s own mental states were coded in responses to question 3 that explicitly prompted reflection on the participant’s own experience. Once the mental state reference was coded as a self-reference, it was further classified into one of three ToM types described in **Table 2**.

#### Controls

Besides gender, and age, we asked participants to indicate the highest level of education they obtained (see **Table 3**). We also included control variables for familiarity with the film scene (yes or no); perceived quality of the film; self-reported proficiency in the English language (from 0 for basic proficiency in writing to 4 for first language); size of screen used; and word count of response to the open ended questions.

**Table 3:** Means and standard deviations for output and other variables.

	Long Shot (Original)		Close-up Neutral		Close-up Sad		Scale
	*M*	*SD*	*M*	*SD*	*M*	*SD*	
ToM-tendency for target	2.29	2.14	1.97	1.52	3.17	2.14	~
ToM-tendency for target (Affective)	1.11	1.60	1.14	1.36	1.86	1.53	~
ToM-tendency for target (Cognitive)	0.74	0.85	0.51	0.90	0.72	0.97	~
ToM-tendency for target (Intentions)	0.43	0.61	0.32	0.53	0.58	0.65	~
ToM-tendency for non-target	1.23	1.68	0.76	0.95	1.28	1.47	~
ToM-ability for target	9.69	4.54	8.81	4.06	10.69	5.13	~
ToM-ability for non-target	0.54	1.01	0.35	0.72	0.36	0.54	~
ToM-self	5.80	3.15	6.21	3.74	6.67	3.84	~
ToM-self (Affective mental states)	3.89	2.35	4.62	3.18	5.08	3.20	~
ToM-self (Cognitive mental states)	1.74	1.75	1.68	1.53	1.39	1.18	~
ToM-self (Intentions)	0.17	0.45	0.05	0.23	0.19	0.47	~
Level of english	1.05	0.22	1.06	0.02	1.00	0.00	(First language) 1 to 5 (basic proficiency)
Highest level of formal education	4.00	1.47	3.76	1.38	3.92	1.42	1 (none), 2 (Secondary education, not completed), 3 (Secondary education, completed), 4 (Trade/Apprenticeship), 5 (Higher cert/Diploma), 6 (Bachelors Degree, 7 (Masters/Ph.D.).
Age	21.83	8.46	23.82	11.09	20.53	5.60	Age in years
Screen size	3.75	0.94	3.87	0.99	3.66	0.91	(Cinema size) 1 to 6 (<than smart phone)
Perceived film quality	4.68	1.60	5.11	1.56	4.63	1.46	(Bad) 1 to 7 (good)

*~Average number of mental state references in a response that was 6 – 10 sentences (in the inferential analysis, Poisson Regression, this was offset against the log transformed word count of the response).*

### Data Analysis

Open responses were coded and group mean scores were calculated separately for target and non-target characters. Data were cleaned, distributions were explored, and descriptive statistics are reported in **Table 3**. Given the nature of the data (count data) the hypotheses were tested using Poisson regression. The independent (predictor) variables were Character depiction condition (Long-shot vs. Sad close-up vs. Neutral close-up), and Character (Target vs. Non-Target). Frequency of mental state references (categorized as ToM-tendency, ToM-ability, and ToM-self) were offset against the log transformed word count in participants’ responses, to account for individual response length in a way that is required for analysis of count data (Agresti, 2003). In addition, to account for the personal relevance of the story, reported gender and age were included as covariates.

## Results

Before testing the hypotheses, a series of one-way ANOVA revealed no significant difference between the experimental groups in their level of English, *F*(2,133) = 1.373, *p* > 0.05, education, *F*(2,109) = 0.266, *p* > 0.05, age, *F*(2,109) = 1.383, *p* > 0.05, or the size of the screen that they viewed the film on, *F*(2,109) = 0.472, *p* > 0.05 (see **Table 3**). Importantly, there was no significant difference observed between the groups in perceived quality of the film, *F*(2,109) = 1.133, *p* > 0.05 demonstrating that the manipulation did not significantly detract from the viewing experience.

### ToM-Tendency

**FIGURE 2:**
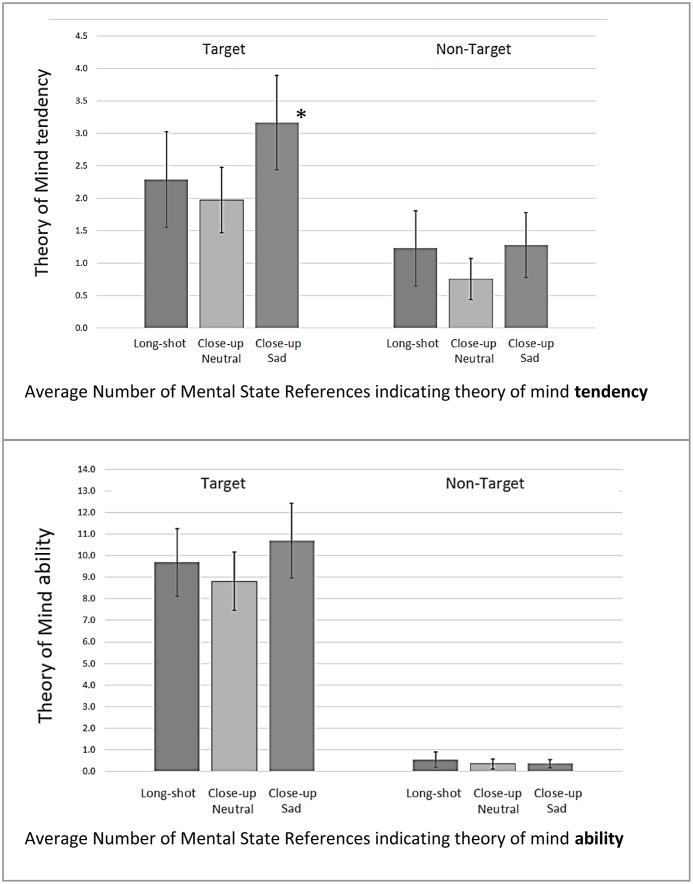
Average number of mental state references indicating theory of mind tendency (top) and ability (bottom) with 95% Confidence Intervals (means shown regardless of word-count). Mental state references are presented categorized by whether they referred to the Target character or Non-target character. The conditions listed on the *X*-axis indicate the way in which the target character was depicted (non-target character was depicted in long shot for each condition). ^∗^ToM-tendency was significantly higher in for the target character when she was presented in close-up with a sad facial expression.

To answer RQ1, we tested how Character depiction (close-up and facial expression) affected participants’ ToM-tendency, and if this differs for the target and non-target character. Analysis revealed a significant interaction between the depiction and the character (target/non-target), *F*(5,214) = 17.43, *p* < 0.01. Results demonstrated that the manipulation affected responses toward the target but not the non-target character (see **Figure 2**). Pairwise contrasts (using least significant difference) demonstrated that participants in the sad close-up condition made significantly more references to target character’s mental states than those in the long-shot condition, *b*(0.053) = 0.104, *p* = 0.05, and the neutral close-up condition, *b*(0.051) = 0.128, *p* = 0.013. This pattern of findings is in line with our prediction that participants in the sad close-up condition would demonstrate the highest level of ToM-tendency, and that it was directed toward the target character’s mental states (rather than the non-target character).

To explore the effect of character depiction further, we tested its effect on the type of mental states for the target character. Results revealed a significant interaction effect between depiction and type of mental state, *F*(8,322) = 7.781; *p* < 0.05. Pairwise comparisons demonstrated that the sad close-up condition was associated with significantly more references to the target character’s affective mental states, than the neutral close-up, *b*(0.047) = 0.095, *p* = 0.045, or long-shot conditions, *b*(0.046) = 0.104, *p* = 0.025. No significant effects of character depiction were evident for the mental state references to cognitions or intentions.

### ToM-Ability

RQ2 explored the effect of character depiction on ToM-ability. While mean levels of mental state references where higher for all conditions in question 2 (which explicitly prompted ToM) compared to question 1, using the same analysis, no significant effects of depiction were observed for the target, *F*(2,214) = 0.38, *p* > 0.05 or for the non-target characters, *F*(2,214) = 1.27, *p* > 0.05 (see **Figure 2**). These results do not support our prediction that the inclusion of close-up shots (especially emotional close-up shots) elicits participants’ ToM-ability toward the target character, and thus hypothesis 2 was not supported.

### ToM-Self

Finally, we tested hypothesis 3 predicting that Character depiction would affect references to one’s own mental states (ToM-self). Results showed a marginally significant effect of depiction on the frequency of ToM-self, χ^2^(4) = 9.16, *p* = 0.057. Relative to the long-shot condition, participants in the neutral close-up condition referred to their own mental states more frequently, χ^2^(1) = 3.137, *Exp(B)* = 1.13; *CI_95_* = [0.987, 1.29]; *p* = 0.077. This effect was even stronger for the sad close-up condition, χ^2^(1) = 3.713, *Exp(B)* = 1.139; *CI_95_* = [0.998, 1.30]; *p* = 0.054, with no significant effect observed between the neutral and sad close-up conditions, χ^2^(1) = 0.023, *Exp(B)* = 0.990; *CI_95_* = [0.870, 1.126]; *p* = 0.879. Thus it seems shot-scale and facial expression affected ToM-self, in line with hypothesis 3.

## Discussion

Using highly controlled yet ecologically valid film stimuli in a true experimental design, we explored the effect of character depiction on viewers’ social cognition. Specifically, we were interested in viewers’ tendency to reference character mental states (ToM-tendency) and their ability to do so when prompted (ToM-ability). Our findings demonstrate that shot-scale and facial expression do affect social cognition. Specifically, we observed that the close-ups of sad faces produced significantly higher ToM-tendency than other conditions, and that the use of a neutral close-up produced no more ToM-tendency than the long-shot version. This suggests that the increase in ToM-tendency response is not driven by merely presenting the character’s face larger in the frame (i.e., at a smaller spatial distance from the viewer), but rather it is the social and emotional information carried by the face that drives ToM-tendency responses. Importantly, this work extends the findings of previous research which demonstrated that exposure to fiction films (as opposed to documentary films) can elicit ToM response (Black and Barnes, 2015) by further exploring the way in which formal features of the narrative can effect types of ToM responding.

Supporting hypothesis 1, the current findings demonstrate an effect of character depiction on participants’ ToM-tendency. More specifically, the close-up shots of the target character with a sad facial expression were associated with higher tendency to refer to the target character’s mental states. Breaking down this finding into the different types of ToM response, we found that the effect was driven by affective ToM. That is, the increase in ToM response primarily consisted of references to the target character’s *affective* mental states, rather than her cognition or intention. The manipulation of facial expression was one of emotional valence; the faces presented were either sad or emotionless. Thus this finding is in line with that of previous research showing that sad expressions elicit affective responses in observers (Knutson, 1996; Hess et al., 2000; Hareli and Hess, 2010). In line with this work, we predict that the ratio of references to the target character’s feelings, thoughts or intentions may change in the context of a different film or if future researchers use different manipulations of facial expression, e.g., a thoughtful face.

An important aim of the present study was to explore whether the ToM-eliciting effect of seeing characters in close-up transfers to character depicted only in long shot (non-target character). Results of the current study showed no effect of character depiction on ToM responding toward the non-target character. Given that the inserted close-up shots did not feature the male character, this is perhaps not surprising. Indeed the characters in the stimulus of the current study differed not only in shot scale but along other dimensions (e.g., gender, age, appearance) which may have also inhibited a transfer effect. Nevertheless it is important because it demonstrates no effect of character depiction on any general form of ToM responses, where previous researchers have reported such general ToM effects using other media formats (e.g., Kidd and Castano, 2013, 2016, 2017; Black and Barnes, 2015; Pino and Mazza, 2016). In line with this, when prompted to recount the narrative events from the perspective of the target character (question 2), all groups demonstrated a higher frequency of mental state references to the target character, with no difference between conditions. This demonstrates that when called upon to do so, there was no difference between groups in terms of participants’ *ability* to mentalize. Thus the use of close-up shots does not increase ToM responding by activating some enhanced mentalizing ability toward all characters, but rather it demonstrates, that close-ups work by directing our attention to the salient aspects of particular characters in the narrative. This is in line with Peskin and Astington’s (2004) findings that adding metacognitive language (words expressing character mental states) into stories improved children’s vocabulary on mental states, but not their performance in a false belief test. It seems that emotional words in printed media have similar function to emotional faces in visual media. Furthermore, filmmakers are skilled in their ability to direct attention toward such important social cues (Loschky et al., 2015; Cutting and Armstrong, 2016).

Character depiction also appears to have affected references to one’s own mental states (ToM-self). Close-ups of sad faces produced higher levels of ToM-self than other conditions. Results show that the neutral close-up condition produced more references to participants’ own emotions than the long-shot condition, and the sad close-up condition produced even more references to participants’ own emotions. These findings show a similar pattern as ToM-tendency responses for the target. They suggest that shot-scale and facial expression do not increase ToM-ability in general, but rather it increases one’s tendency to mentalize toward the target, and in doing so may facilitate identification of their own mental states. This finding is in line with the large body of research linking the processes of social cognition of others, with self (e.g., Vygotsky, 1978; Neisser, 1988; Gallese, 2003) and the evidence for overlapping neural mechanisms in these processes (Decety and Jackson, 2004; Gallese, 2007; Lieberman, 2007; Rooney et al., 2012). Drawing on this work, we argue that directing attention to others’ mental states, aids recognition of one’s own mental states.

### Synthesis

Taken together, the findings have implications for our understanding of the nature of ToM responses toward characters. They demonstrated that viewers did not differ in their ToM-ability, but rather they differed in their ToM-tendency. Showing the sad facial expression of a fictional character makes viewer mental states more readily available and featured more in their unprompted responses. But when prompted, all groups demonstrated the ability to call on social cognitive faculties to model the characters’ mental states. These findings have important implications for the way in which ToM responses are measured in future research studies, and how they have been measured in the past. Here we show the way in which participants are asked about the experience can have a large impact on the findings. Accessing unprompted ToM responses may show differences that are not evident in prompted responses. This is particularly important given that so many ToM measures use direct questions to assess participants’ ability to mentalize, rather than observing their uncontaminated responses. The failure to distinguish between these aspects of ToM may explain why previous research has presented conflicting and ambiguous results (e.g., Kidd and Castano, 2013, 2016, 2017; Panero et al., 2016; Pino and Mazza, 2016). In line with researchers such as Apperly (2012), Meins et al. (2014), and Rosenblau et al. (2015), we argue that capturing unprompted ToM responses taps in to participant’s ToM-tendency and is representative of how ToM manifests in everyday life. Thus we too, call on researchers to give careful consideration to the operational definitions of social cognition they employ and the claims that can be made from their findings.

### Limitations and Implications for Future Work

The strength of our own claims is somewhat limited by our focus on a single emotion manipulation, in a single film stimulus. Indeed the stimulus used was an animation rather than live action. This means that our findings presented in the context of simple highly designed visual information and call on future research to extend the findings with even more ecological validity. Nevertheless, we argue that this is an important strength of our work too. The stimulus used (its design and manipulation) offers a degree of experimental control that is typically difficult to achieve, without contaminating the ecological validity of the study. This major strength of the current study, compliments previous research that explored the relationship between ToM responses and shot-scale distribution in different films (Bálint et al., 2016). Taken together these studies, using various films (Bálint et al., 2016) and in a single experimentally manipulated film (the present study) provide evidence that the distribution of close-up shots may be utilized to increase ToM responding. Importantly, here we do not propose that simply inserting close-up shots into film will automatically generate increased ToM responses in viewers. Indeed, our findings that show an effect for the facial expression demonstrate that the social information presented in the close-up is particularly important in directing attention toward character mental states. In addition, we recognize that other ways in which the close-up is used will drive the ToM responses. Future research needs to explore these subtleties further by, for example, manipulating the number and position of the close-ups used, or how the depiction of the character might interact with viewer identity or personal relevance of the narrative.

We propose that using close-up shots of a sad expression drew participants’ attention to the character’s mental states, made character mental states more accessible and thus more likely to be integrated into viewers’ models of the narrative. To be clear, we make this proposal for the current sample, and those within a population that they represent. The current sample of participants where relatively young adults in university education and our findings demonstrated that when eventually prompted to take the perspective of the target character, all groups regardless of condition, were able to do so. It is clear that the nature of our sample (convenient sample of volunteers) limits the extent to which the findings might generalize. While we stand by the way in which these findings speak to previous research, with similar limitations, we expect future research to build upon this limitation and design novel ways in which data can be collected (ethically) from a more representative and diverse population. For example, it remains to be seen how these findings may be extended to populations with deficits in social cognition such as participants with autism or schizophrenia. These populations may not be able to mentalize when prompted to do so. We might speculate that simply inserting close-ups would not increase ToM responding for an autistic population without some form of guidance or scaffolding, i.e., additional resources to draw attention to relevant social information.

## Conclusion

Using a true experimental design, with highly controlled visual stimuli in an ecologically valid activity, the present study makes an important contribution to our understanding of theory of mind response. The findings indicate that depiction of the character can direct attentional focus toward their mental states, making them more accessible to the viewer and thus increasing viewers’ *tendency* to use those mental states in a representation of the narrative. However, mere exposure to close-up faces of characters does not enhance general theory of mind *ability*, nor does it transfer to mentalizing with other characters depicted in long shots. Finally, the findings demonstrate that directing viewers’ attention to the mental states of characters also elicits viewers’ modeling of their own mental state, supporting the idea that understanding mental states in others is linked to understanding self. Findings of the present study show that shot scale and facial expression of character depiction is a powerful tool for shaping viewers’ recognition of mental states in characters on screen and in self.

## Ethics Statement

The current study was considered by the host institution’s ethics review board and deemed exempt from full review due to the low risk involved. All participants read the full information sheet and gave electronic consent to participate. Participation was online, so no individual participant was directly canvassed and all were free to discontinue anonymously.

## Author Contributions

BR and KB made equal and substantial contributions to the conception and design of the work, the acquisition, analysis, and interpretation of data for the work. BR and KB drafted the work and critically revised it for important intellectual content. BR and KB made a final approval of the version to be published. BR and KB agree to be accountable for all aspects of the work in ensuring that questions related to the accuracy or integrity of any part of the work are appropriately investigated and resolved.

## Conflict of Interest Statement

The authors declare that the research was conducted in the absence of any commercial or financial relationships that could be construed as a potential conflict of interest.
