# Corrigendum: Watching More Closely: Shot Scale Affects Film Viewers' Theory of Mind Tendency But Not Ability

**DOI:** 10.3389/fpsyg.2018.00261

**Published:** 2018-03-01

**Authors:** Brendan Rooney, Katalin E. Bálint

**Affiliations:** ^1^School of Psychology, University College Dublin, Dublin, Ireland; ^2^Tilburg Center for Cognition and Communication, Tilburg University, Tilburg, Netherlands; ^3^Institute for Media, Knowledge and Communication, University of Augsburg, Augsburg, Germany

**Keywords:** theory of mind, shot scale, close up shot, facial expression, characters, film

In the original article, we referred to Canini et al., 2013. This was an error. It should be Canini et al., 2011. In the reference section the reference was incorrectly written as:

Canini, L., Benini, S., and Leonardi, R. (2013). Classifying cinematographic shot types. *Multimed. Tools Appl*. 62, 51–73. doi: 10.1007/s11042-011-0916-9

The correct reference should be:

Canini, L., Benini, S., and Leonardi, R. (2011). “Affective analysis on patterns of shot types in movies,” in *Proceedings of the 7th International Symposium on Image and Signal Processing and Analysis (ISPA 2011)*.

The authors apologize for this error and state that this does not change the scientific conclusions of the article in any way.

The original article has been updated.

## Conflict of interest statement

The authors declare that the research was conducted in the absence of any commercial or financial relationships that could be construed as a potential conflict of interest.

